# Investigating The Alterations of Oxidative Stress Status, Antioxidant Defense Mechanisms, MAP Kinase and Mitochondrial Apoptotic Pathway in Adipose-Derived Mesenchymal Stem Cells from STZ Diabetic Rats

**DOI:** 10.22074/cellj.2020.6958

**Published:** 2020-09-08

**Authors:** Azadeh Aminzadeh, Neda Tekiyeh Maroof, Mehrnaz Mehrabani, Kobra Bahrampour Juybari, Ali Mohammad Sharifi

**Affiliations:** 1. Department of Pharmacology and Toxicology, School of Pharmacy, Kerman University of Medical Sciences, Kerman, Iran; 2. Razi Drug Research Center, Department of Pharmacology, School of Medicine, Iran University of Medical Sciences, Tehran, Iran; 3. Physiology Research Center, Institute of Basic and Clinical Physiology Sciences, Kerman University of Medical Sciences, Kerman, Iran; 4. Department of Pharmacology, Semnan University of Medical Science, Semnan, Iran; 5. Department of Tissue Engineering and Regenerative Medicine, School of Advanced Technologies in Medicine, Iran University of Medical Sciences, Tehran, Iran

**Keywords:** Adipose-Derived Stem Cells, Antioxidant, Apoptosis, Cell Therapy, Diabetes

## Abstract

**Objective:**

This study aimed to investigate the reliability of diabetic adipose-derived stem cells (ADSCs) for autologous cell-based therapies by exploring the functionality of signalling pathways involved in regulating oxidative stress and apoptosis.

**Materials and Methods:**

In this experimental study, ADSCs were isolated from streptozotocin (STZ)-induced diabetic rats (dADSCs) and normal rats (nADSCs). The colonies derived from dADSCs and nADSCs were compared by colony-forming unit (CFU) assay. Reactive oxygen species (ROS) formation and total antioxidant power (TAP) were also measured. Furthermore, the expression of antioxidant enzymes, including catalase (Cat), superoxide dismutase (Sod)-1 and -3, glutathione peroxidase (Gpx)-1, -3 and -4 was measured at mRNA level by semi-quantitative reverse transcriptase polymerase chain reaction assay. The expression of Bax, Bcl2, caspase-3, total and phosphorylated c-Jun N-terminal kinase (JNK) and P38 Mitogen-Activated Protein Kinase (MAPK) at protein level was analyzed by western blotting.

**Results:**

The results of this study indicated that viability and plating efficiency of dADSCs were significantly lower than those of nADSCs. ROS generation and TAP level were respectively higher and lower in dADSCs. The gene expression of antioxidant enzymes, including Cat, Sod-1, Gpx-3 and Gpx-4 in dADSCs was significantly greater than that in nADSCs. However, Sod-3 and Gpx-1 mRNA levels were decreased in dADSCs. Moreover, Bax/Bcl-2 protein ratio, caspase-3 protein expression and phosphorylation of JNK and P38 proteins were increased in dADSCs compared to nADSCs.

**Conclusion:**

Taken together, diabetes might impair the cellular functions of dADSCs as candidates for autologous cell- based therapies. This impairment seems to be mediated by JNK, P38 MAPKs, and mitochondria pathway of apoptosis and partly by disruption of antioxidant capacity.

## Introduction

In type 1 diabetes, destruction of beta cells located in the pancreas, leads to elevated blood glucose levels and pathological changes in different organs (1). Currently, insulin therapy is the most common therapeutic strategy used for type 1 diabetes. Since, in some cases, insulin therapy could not properly control the progression of diabetes and its complications (2), other alternative therapies might be desirable (3, 4). Modern therapeutic approaches not only mitigate the symptoms of the disease but also improve organs’ function. Stem cell therapy has been proposed as a promising therapeutic strategy for a number of degenerative disorders including type 1 diabetes (5).

Adipose derived stem cells (ADSCs) exhibit the ability to self-renew and differentiate into various functional cell types. Moreover, ADSCs have higher availability, require minimal invasive harvesting procedure and exert a larger yield as compared with other mesenchymal stem cells. Therefore, ADSCs are known as suitable candidates for cell therapy (6).

Oxidative stress is considered to play an important role in the development of various diseases including diabetes mellitus. Free radicals like superoxide radical (O_2_^-^) that are abundantly produced under diabetic conditions, may hamper the normal function of mesenchymal stem cells (7, 8). For example, it was shown that diabetes impeded the growth and differentiation ability of bone marrow mesenchymal stem cells (BMSCs) (9) and mitigated the angiogenic potential of ADSCs (10). In normal situations, natural enzymatic and non-enzymatic antioxidant defense system protects the body’s organs against free radical damage. Free radicals could be eliminated by antioxidant enzymes, such as catalase (CAT), superoxide dismutase (SOD) and glutathione peroxidase (GPx) (11) which were shown to be affected by diabetes (12). There is controversial data in the literature regarding the effects of diabetes on antioxidant enzymes (13) that may be due to differences between organs or experimental conditions like the duration of diabetes induction (14, 15). Moreover, the involvement of mitogen-activated protein kinases (MAPKs) and Bcl-2 family in diabetes progression, was shown (16). MAPKs include c-Jun NH2-terminal kinases (JNK), P38 and extracellular signal-regulated kinase1/2 (ERK1/2) and play key roles in cell viability and death (17). The Bcl-2 family is engaged with apoptosis and survival control, and Bax and Bcl-2 are known as pro- and anti-apoptotic proteins, respectively (18). However, the effect of diabetes on MAPKs, Bcl-2 family and antioxidant enzymes in ADSCs, is still unknown. Therefore, the aim of the current study was to investigate diabetes-induced impaired mechanisms, in particular those related to regulation of oxidative stress and apoptosis, in ADSCs.

## Materials and Methods

Fluorescein isothiocyanate (FITC)-conjugated CD90 and CD45 antibodies and puri.ed anti-rat antibody to CD73 were purchased from BD Biosciences Pharmingen (San Diego, CA, USA). FITC-conjugated CD11b antibody was bought from GeneTex, Inc. (USA). FITC-conjugated CD31 and 44 antibodies were purchased from Serotec (Oxford, UK). Bax and Bcl-2 antibodies were bought from Abcam (Cambridge, UK). Horseradish peroxidase (HRP)-conjugated secondary antibody, caspase-3, β-actin, c-Jun N-terminal kinase (JNK), P38, Phospho- JNK and P38 antibodies were obtained from Cell Signaling (Danvers, MA, USA). 2, 4, 6-tri (2-pyridyl)-striazine (TPTZ) was bought from Merck (Germany) and 3-(4, 5-dimethylthiazol-2-yl)-2, 5-diphenyl tetrazolium bromide (MTT), streptozotocin (STZ), collagenase type I and 2, 7-dichlorofluorescein diacetate (DCF-DA) were bought from Sigma (Sigma Aldrich, St. Louis, MO, USA). All cell culture materials were obtained from Gibco (Carlsbad, CA, USA).

### Animals, study design and induction of diabetes

In this experimental study, young male Wistar rats (200- 250 g) were obtained from Pasteur Institute, Tehran, Iran and kept at room temperature (22 ± 2 °C) with humidity of 45-55% and 12:12 hours light-dark cycle. The experiment was conducted in accordance with the guidelines of the Ethics Committee of Iran University of Medical Sciences, based on National Institutes of Health Principles of Laboratory Animal Care (NIH Publications No. 8023, revised 1978). Two equal groups were randomly selected from 20 male Wistar rats. Control group was injected with a single intra-peritoneal injection of normal saline as solvent of STZ. Diabetic group was injected with a single intra-peritoneal injection of STZ (55 mg/kg) dissolved in normal saline. Blood glucose level was monitored 7 days later. Only rats with blood glucose levels of >300 mg/dl were considered diabetic and kept for four months. During this period, blood glucose levels were measured monthly. Levels of haemoglobin A1C (HbA1c) were tested in rats before being euthanized and only rats with an HbA1c≥ 6.5 were included.

### Isolation and culture of adipose-derived stem cells

Rats were euthanized using ketamine/xylazine overdose and then, adipose tissues were obtained from epididymal fat pads of rats. Adipose tissues were minced, washed extensively in PBS (Sigma Aldrich, St. Louis, MO, USA) containing 5% antibiotic (100 U/ml penicillin and 100 μg /ml streptomycin) and digested using 0.075% collagenase I (prepared in PBS) at 37°C for 30 minutes. After adding α-modified Eagle’s medium (α-MEM), containing 10% fetal bovine serum (FBS, Gibco, Carlsbad, CA, USA) to neutralize collagenase, cell suspension was centrifuged at 2000 rpm for 10 minutes and the supernatant was discarded. Pellet was washed with PBS and filtered using 100 μm nylon mesh. Then, filtered fraction was centrifuged at 2000 rpm for 10 minutes and the pellet was incubated at 37°C in 5% CO_2_ with 95% humidity in the growth medium (α-MEM with 20% FBS and 100 U/ml penicillin and 100 μg /ml streptomycin). After 72 hours, non-adherent cells were removed and the growth medium was changed every 72 hours. All ADSCs used in this study, were from passage 3-4 (10).

### Characterizations of adipose-derived stem cells

To characterize the phenotypes of the isolated ADSCs, the cultured ADSCs were harvested and stained with antibodies against CD44, CD73, CD90, CD45, CD11b and CD31. Cell phenotyping was performed using flow cytometry analysis by a FACS caliber cytometer (Becton Dickinson, San Diego, CA, USA).

### Colony-forming unit assay

To compare the number of nADSCs- and dADSCsderived colonies, CFU test was performed. Briefly, cells at early passages were cultured in 6-well plates. Two weeks later, all wells were washed with PBS and 5 ml of crystal violet solution (0.5% prepared in methanol) was added. After 30 min, wells were washed with PBS, and the numbers of colonies with a minimum of 50 cells were assessed using NIH Image J software (http://rsbweb.nih.gov/ij). Then, plating efficiency was calculated according to the following formula:

Plating efficiency=The number of colony /number of cells plated×100.

### MTT and propidium iodide assay

Propidium iodide (PI) staining and MTT assays were used to assess apoptosis and cell prolifration rate, respectively. dADSCs and nADSCs cells were seeded in 96-well plates. After 24, 48 and 72 hours, cells were exposed to MTT dye for 4 hours. Subsequently, the media were discarded, and dimethyl sulfoxide (DMSO) was added. The absorbance was measured at 570 nm using a micro-plate reader. Cell proliferation rate was measured as percentage of control. In PI method, the cells were harvested and fixed in ethanol (70%) on ice for 4 hours. The cells were treated with RNase and incubated with PI staining solution. The stained cells were analyzed by flow cytometry (Becton and Dickinson Co., USA) (7).

### Measurement of intracellular reactive oxygen species

DCFH-DA is a lipophilic compound that can pass through the plasma membrane. Intracellular ROS oxidizes non-fluorescent DCF-DA to the highly fluorescent 2′-7′-dichlorofluorescein (DCF). Briefly, nADSCs and dADSCs were harvested in 24-well plates for 72 hours. After discarding media and rinsing cells with PBS, ADSCs were incubated with DCF-DA for 30 minutes. For the last time, cells were washed with PBS and medium was added. Then, the absorbance of DCF was assessed at 485/20 (nm) excitation and 528/20 (nm) emission wavelength using a multi-detection microplate reader.

### Measurement of total antioxidant power

In this assay, reduction of ferric tripyridyltriazine to a blue ferrous complex by samples, is considered an indicator of TAP. At first, 290 μl of fresh working solution (25 ml acetate buffer (300 mM), 2.5 ml TPTZ (2, 4, 6 tripyridyl-s-triazine) solution (10 mM in 40 mM HCl), and 2.5 ml FeCl_3_.6H_2_O) was added to 10 μl of cells supernatants. The mixture was incubated at 37°C for 10 minutes and the absorbance of the blue complex between Fe^2+^ and TPTZ, was read at 593 nm (Bio-Tek ELX800, USA) (19).

### Western blot analysis

Firstly, dADSCs and nADSCs were exposed to 1X RIPA lysis buffer that contained 10 μl protease and phosphatase inhibitor cocktail. The resultant mixture was centrifuged for 20 min at 15,000 g at 4°C. Total protein concentration was determined by Bradford method (20). The supernatant was stored at -80°C. Equal amount of samples (70 μg) was separated on 10-12% sodium dodecyl sulfate polyacrylamide gel electrophoresis (SDS-PAGE) gel. The proteins were transferred to a polyvinylidene fluoride (PVDF) membrane. It was followed by incubation with primary antibodies overnight at 4°C and then, HRPlabeled secondary antibody for 1 hour. An enhanced chemiluminescence kit (Amersham Pharmacia Biotech, NJ, USA) was used to visualize the protein bands. β-actin was used as the internal control. Then, band density was quantified by total Lab software (UK) (21).

### Semi-quantitative reverse transcriptase polymerase chain reaction

Total RNA was extracted from the nADSCs and dADSCs using *RNX*-PLUS kit (*Cinnagen*, Iran) according to the manufacturer’s instructions. Afterwards, cDNA was synthesized using RNA (1 μg), oligo-dT primer (2 μg, Fermentase, USA) and MMLV (200 U, Fermentase, USA) in a total volume of 20 μl. Reaction was done at 42˚C for 1 hour, and continued at 72˚C for 10 minutes. Finally, polymerase chain reaction (PCR) reaction was done using cDNA (5 μl) and specific primers (Table 1). *β-actin* was used as the internal control. Final products were run on agarose gels (2%) and stained with Nancy-2*50*. Then, band was quantified by total lab software (UK).

**Table 1:** Primers used for semi-quantitative reverse transcriptase polymerase chain reaction


Gene	Primer sequence (5ˊ-3ˊ)	Product size (bp)

*Cat*	F: GGTAACTGGGACCTTGTGGG	222
	R: GCCATTCATGTGCCGATGTC	
*Sod1*	F: AAGCGGTGAACCAGTTGTGG	187
	R: ATTGCCCAGGTCTCCAACAT	
*Sod3*	F: GCTTGTCAGGTGTGGAACC	172
	R: CAGGTCTTTGGAGTGCGTG	
*Gpx1*	F: ACCGTGTATGCCTTCTCC	221
	R: TTGCCATTCTCCTGATGTCC	
*Gpx3*	F: ACCATCTGTGCTCACGGTTT	187
	R: GAAGGAGGTGGTGGCATAG	
*Gpx4*	F: GGAGCCCCAGGTGATAGAG	137
	R: CTGGTTTTCAGGCAGACCGT	
*β-actin*	F: TGTCCACCTTCCAGCAGATGT	101
	R: AGCTCAGTAACAGTCCGCCTAGA	


### Statistical analysis

Data are presented as the mean ± S.E.M. Data were analyzed by unpaired student’s t test for comparisons between two groups. A P<0.05 was considered statistically significant. All data were analyzed by Graphpad Prism 5.0.

## Results

### Flow cytometry analysis of CD markers

**Fig.1:**
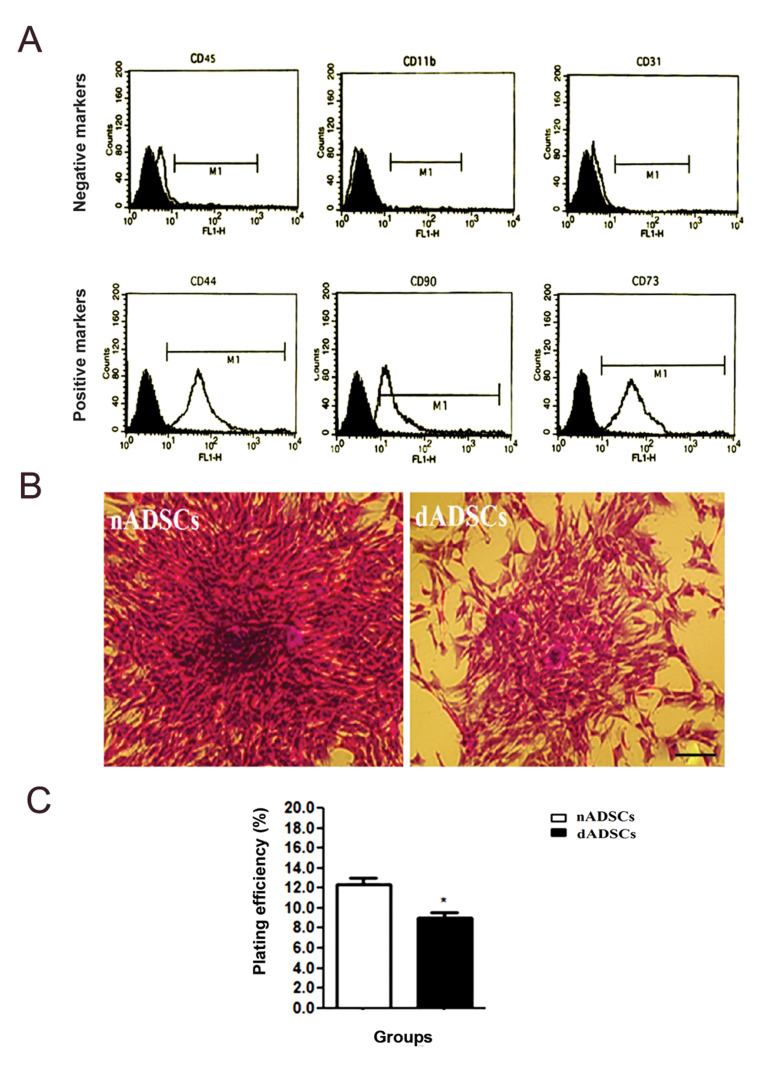
Identification of ADSCs and assessment of their plating efficiency. **A.** Flow cytometry analysis showed that surface markers including CD44, CD73 and CD90 were positive. While CD45, CD11b and CD31 were negative.** B.** Representative images of colonies derived from nADSCs and dADSCs, obtained by light microscopy. **C.** Assessment of plating efficiency of nADSCs and dADSCs. Results are shown as the mean S.E.M (n=3). *; P<0.05 shows significant differences versus control, nADSCs; Normal adipose-derived mesenchymal stem cells, and dADSCs; Diabetic adipose-derived mesenchymal stem cells.

Phenotype of nADSCs or dADSCs was confirmed by flow cytometry analysis. The cell surface markers including CD44, CD73 and CD90, were positive while CD45, CD11b and CD31 were negative. These results indicated that the cultured cells could be considered mesenchymal stem cells (Fig .1A).

### Effect of diabetes on plating efficiency of dADSCs and nADSCs

CFU assays demonstrated that plating efficiency of dADSCs (9 ± 0.57) was remarkably lower than that of nADSCs (12.33 ± 0.66, P<0.05, Fig .1B, C).

### Effect of diabetes on cell proliferation rate and apoptosis

**Fig.2:**
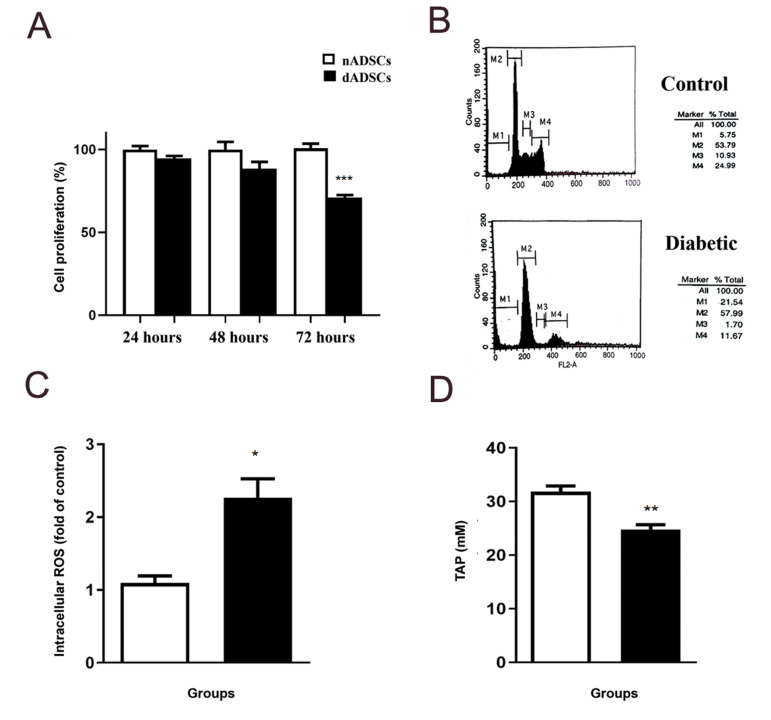
Effect of diabetes on proliferation rate, apoptosis and antioxidant status of ADSCs. **A.** Effect of diabetes on proliferation rate of ADSCs. **B.** Effect of diabetes on apoptosis rate of ADSCs; Sub-G_1_, G_1_, S and G_2_/M phases were separated by gates M1, M2, M3 and M4, respectively. **C.** ROS formation and **D.** Total antioxidant power. Results are shown as the mean S.E.M (n=3). *; P<0.05, **; P<0.01, and ***; P<0.001 Show significant differences versus controls, ADSCs; Adipose-derived stem cells, and ROS; Reactive oxygen species.

nADSCs and dADSCs were cultured for 24, 48 and 72 hours and proliferation rate was measured. As shown in Figure 2A, the proliferation rate of dADSCs was significantly lower when compared with nADSCs after 72 hours incubation (P<0.001). To determine the effects of diabetes on cell apoptosis, PI staining was conducted. As shown in Figure 2B, diabetes pushed cells to commit apoptosis.

### Effect of diabetes on intracellular reactive oxygen species and total antioxidant power level

As shown in Figure 2C, diabetes significantly increased DCF fluorescence as compared with the control group (P<0.05). Figure 2D shows that TAP value decreased in the diabetic group as compared with the control group (P<0.01).

### Effects of diabetes on Bax and Bcl-2 protein levels and caspase-3 activation

**Fig.3:**
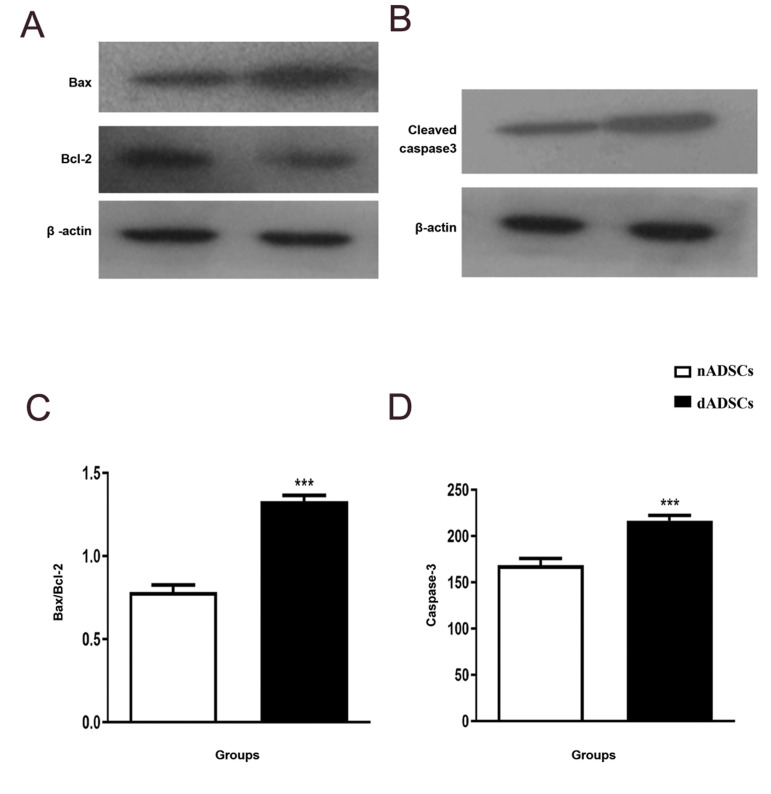
Effects of diabetes on the Bax/Bcl-2 proteins ratio and cleaved caspase-3 protein. Effects of diabetes on the **A. **Bax/Bcl-2 proteins ratio, and **B.** Cleaved caspase-3 protein in nADSCs and dADSCs. Results are shown as the mean ± SEM (n=3). ***; P<0.001 Shows significant differences versus controls, nADSCs; Normal adipose-derived mesenchymal stem cells, and dADSCs; Diabetic adiposederived mesenchymal stem cells.

The protein expression of Bax and Bcl-2 and cleaved caspase-3 was measured by western blotting. As shown in Figure 3A, the results revealed that Bax/Bcl-2 ratio in dADSCs was significantly greater than that in nADSCs (P<0.001). Furthermore, as previously shown, active form of caspases is produced by proteolytic cleavage (22). Hence, we examined cleaved caspase-3 protein expression. Results indicated that cleaved caspase-3 levels markedly increased in dADSCs as compared with nADSCs controls (P<0.001, Fig .3B).

### Effects of diabetes on JNK and P38 MAPKs protein phosphorylation

**Fig.4:**
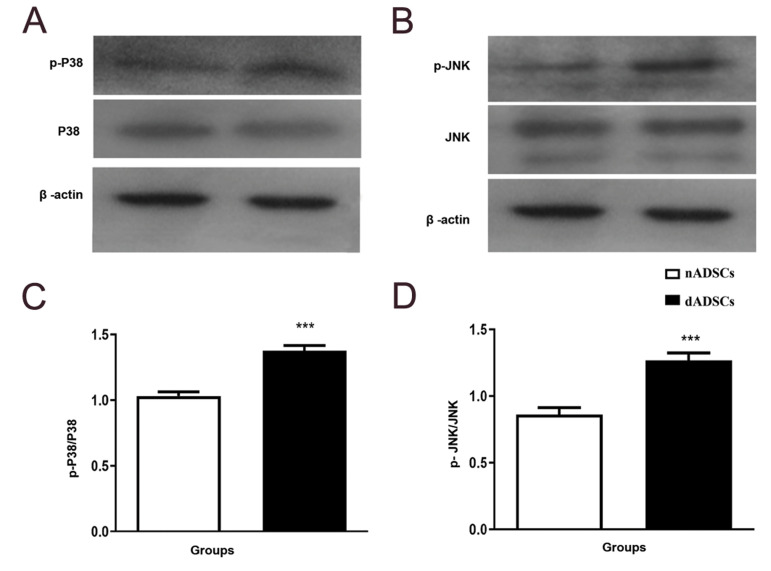
Effects of diabetes on phosphorylation of P38 and JNK MAPKs in nADSCs and dADSCs. **A.** The density of phosphorylated and total P38 were determined and the ratio was calculated. **B.** The density of phosphorylated and total JNK were determined and the ratio was calculated. Results are shown as the mean ± SEM (n=3). ***; P<0.001 shows significant differences versus controls, nADSCs; Normal adipose-derived mesenchymal stem cells, and dADSCs; Diabetic adipose-derived mesenchymal stem cells.

Western blotting was performed to determine whether dADSCs had different expression of JNK and P38 compared to nADSCs controls. Current results revealed that phosphorylated JNK and P38 were significantly increased in dADSCs as compared with the controls (P<0.001). Total JNK and P38 protein levels did not differ between the two groups (Fig .4).

### Effect of diabetes on mRNA levels of antioxidant enzymes

**Fig.5:**
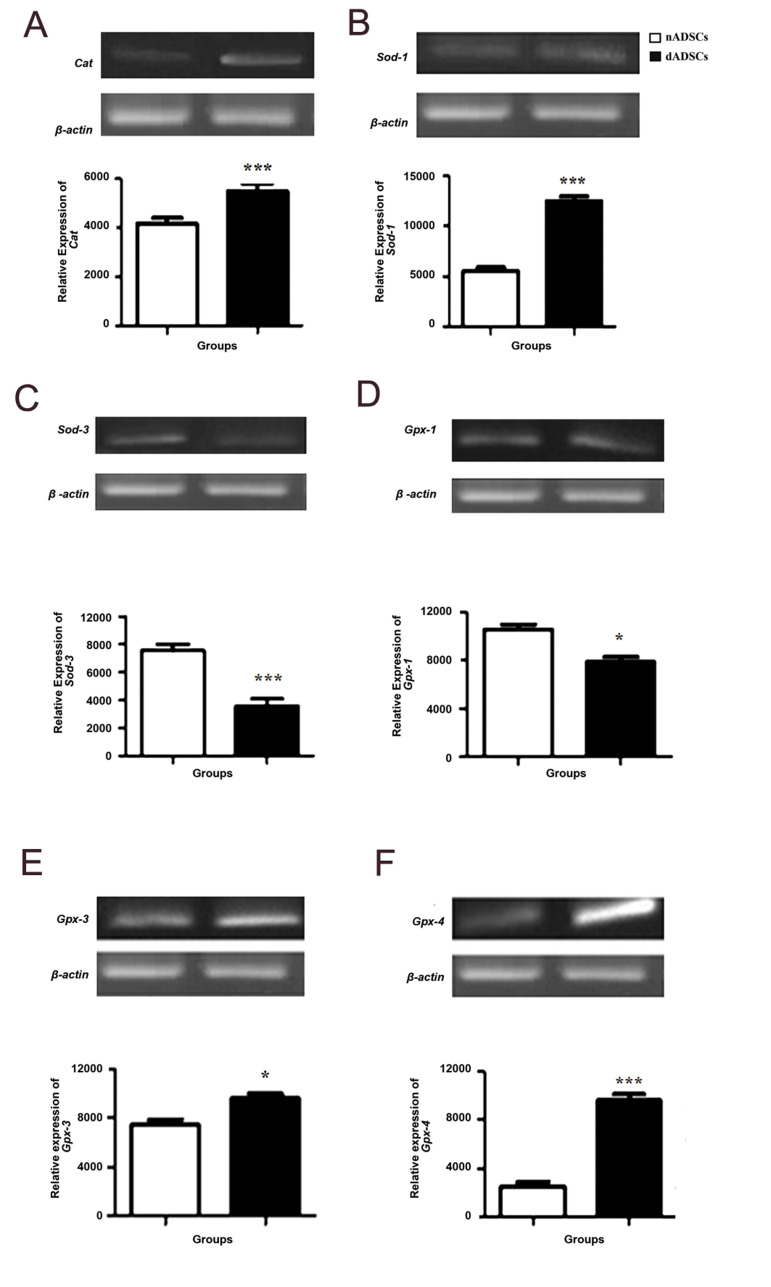
Effects of diabetes on relative mRNA expression level of antioxidant enzymes. Effects of diabetes on relative mRNA expression level of **A.** *Cat*, **B. ***Sod-1*, **C.** *Sod-3*, **D.** *Gpx-1*,** E.** *Gpx-3*, and **F.** *Gpx-4* in the dADSCs in comparison with the control group. Results are shown as the mean ± SEM (n=3). *; P<0.05, ***; P<0.001 show significant differences versus controls, and dADSCs; Diabetic adipose-derived mesenchymal stem cells.

Antioxidant enzymes mRNA levels were measured by sqRT-PCR using gene-specific primers (Table 1). A significant decrease in *Sod-3* (P<0.001) and *Gpx-1* (P<0.05) mRNA levels was observed in dADSCs as compared with the control group. Furthermore, a significant increase in *Cat* (P< 0.001), *Sod-1* (P<0. 001), *Gpx-3* (P<0.05) and *Gpx-4* (P<0.001) mRNA levels was detected in dADSCs in comparison with the control group (Fig .5).

## Discussion

The current study showed that proliferation rate and plating efficiency of dADSCs was considerably lower in comparison with nADSCs. Furthermore, ROS generation and TAP level in dADSCs were respectively higher and lower compared to those of nADSCs. The gene expression of antioxidant enzymes including *Cat*, *Sod-1*, *Gpx-3* and *Gpx-4* was significantly increased in dADSCs as compared with nADSCs. In contrast, *Sod-3* and *Gpx-1* mRNA levels were decreased in dADSCs. Bax/Bcl-2 protein ratio, cleaved caspase-3 protein and phosphorylation rate of JNK and P38 proteins were increased in dADSCs as compared with nADSCs.

Recently, stem cell therapy has been proposed as an appropriate alternative approach for treatment of various disorders including diabetes. However, diabetes could induce many changes at molecular levels in cells including stem cells causing serious functional impairment (10). Therefore, there is a need to identify intrinsic mechanisms disrupted by diabetes. Current study investigated the effect of diabetes-induced alterations in ADSCs isolated from diabetic rats to elucidate some signalling pathways involved in oxidative stress and consequently mitochondrial apoptosis.

Our results indicated that proliferation rate of dADSCs was lower than that of non-diabetic controls and apoptotic cell death in dADSCs was higher compared to nADSCs. This is in agreement with our previous report which demonstrated that diabetic stem cells showed significantly lower proliferation (10) rate. Furthermore, a higher apoptosis rate was shown for diabetic stem cells as compared with controls (23). In contrast, there is a report indicating similar proliferation rate in dADSCs and nADSCs of mice (24). However, differences between experimental conditions like duration of diabetes induction may cause this discrepancy.

It was also shown that higher ROS levels in diabetes may be linked to cell senescence and death (23). Excess production of ROS, an important apoptotic mediator, can cause organ injuries (25). Our results showed that ROS concentration was elevated in dADSCs. In agreement with our findings, a previous study showed that oxidative stress leads to impaired self-renewal of adult stem cells* in vitro* (26).

Under oxidative stress conditions, anti-oxidative enzymes such as CAT, SOD, and GPx act as a defense barrier against oxidative damages (27). Hydrogen peroxide is quickly reduced to superoxide by SOD which can be detoxified by activities of GPx and CAT (28). In this study, we found that total antioxidant capacity reduced in diabetic ADSCs, whereas there were various alterations in antioxidants enzyme mRNA expression. This result may emphasize the fact that the expressions of some of these enzyme have been elevated in response to oxidative stress in a compensatory manner to protect against the insult to the cells. In agreement with this result, a previous study demonstrated that mRNA levels of renal *Sod-1*, *Cat* and *Gpx* of diabetic rats enhanced as compared with controls (29). In opposite, a previous study performed on diabetic heart, showed that total SOD and total GPx expression/ activity were declined whereas CAT expression/activity were enhanced (30). Moreover, another study conducted on the brain tissue demonstrated a significant decrease in mRNA transcription levels of *Sod*, *Cat*, and *Gpx* in the diabetic group compared to the controls (31).

Our results indicated that diabetes affects apoptotic pathways in dADSCs. In intrinsic pathway, Bax dimmers form mitochondrial membrane pores increase membrane permeability allowing the release of pro-apoptotic factors. Bcl-2 could form heterodimers with Bax, preventing oligomerization and pore assembly in mitochondrial membrane (32). Our results revealed that Bax/ Bcl-2 ratio significantly augmented in dADSCs. In line with these results, we previously demonstrated similar results indicating that high glucose levels can cause apoptosis through elevating Bax/Bcl-2 ratio in PC12 cells (18). Another study showed that in BMSCs from patients with systemic lupus erythematous, the levels of Bcl-2 expression were significantly decreased while the Bax expression was remarkably increased as compared with normal controls (18, 33). As final stage of apoptosis, caspase-3 is activated to induce DNA fragmentation and cell death (34). Our results demonstrated that diabetes could elevate the expression of caspase-3 as an ultimate determinant of apoptosis. A former study on PC12 cells demonstrated that high glucose levels increased the expression of caspase-3, caspase-8 and caspase-9 (35).

In addition to Bcl2 family and caspase, MAPKs containing JNK and P38 are important regulators of apoptosis. It was indicated that activation of JNK and P38 is involved in apoptosis induced by various cellular stresses (36). Moreover, it was shown that hyperglycemia could activate JNK and p38 in PC12 neuronal cell (37). Oxidative stress and apoptosis might be inhibited by preventing JNK and p38 phosphorylation. We demonstrated that p-JNK/JNK and p-P38/P38 were increased in dADSCs. This finding is in agreement with a previous study demonstrating a higher level of expression and activation of JNK and p38 in sural nerve of type I and II diabetic patients (38). Taking together, our results suggested that dADSCs showed an overall enhancement in various apoptotic pathways as compared with the controls. It was also suggested that oxidative stress and advanced glycation end products (AGES) are major factors contributing to alterations in MSCs (39).

To explain how this impairment could affect cellular function and lead to complications in diabetes, it is important to realize that, normal MSCs are present in various tissues and have the potential to differentiate into multiple cell types, playing a central role in maintaining tissue homeostasis under both physiological and pathological conditions (40). Moreover, it was previously shown that autologous ADSCs have the ability to secret many potent angiogenic factors including vascular endothelial growth factor (VEGF), fibroblast growth factor (FGF), and insulin growth factor-1 (IGF-1) and may also play an important role in enhancement of angiogenesis and anti-apoptotic ability in diabetes (9, 10). Consequently, loss of action or functional impairment of dADSCs may be responsible for some of the diabetes complications.

## Conclusion

It could be concluded that hyperglycemia might stimulate oxidative stress, resulting into ADSCs damage. Although the current study revealed a significant increase in most of mRNA levels of antioxidant enzymes in ADSCs from STZ-induced diabetic rats, the total antioxidant capacity reduced in these cells. It may be suggested that this compensatory response may no longer survive and hyperglycemia could induce apoptosis leading to impaired cellular functions causing ADSCs death. It may also be concluded that dADSCs may be unsuitable stem cell sources for cell therapy and it may also be speculated that other diabetes complications may possibly be associated with some of these impaired mechanisms.
